# A Practical Guide to Relugolix: Early Experience With Oral Androgen Deprivation Therapy

**DOI:** 10.1093/oncolo/oyad036

**Published:** 2023-03-08

**Authors:** Saro Kasparian, Oren Wei, Ni-Chun Tsai, Joycelynne Palmer, Sumanta Pal, Yung Lyou, Tanya Dorff

**Affiliations:** Department of Medical Oncology, City of Hope Comprehensive Cancer Center, Duarte, CA, USA; Department of Medical Oncology, City of Hope Comprehensive Cancer Center, Duarte, CA, USA; Department of Biostats, City of Hope Comprehensive Cancer Center, Duarte, CA, USA; Department of Biostats, City of Hope Comprehensive Cancer Center, Duarte, CA, USA; Department of Medical Oncology, City of Hope Comprehensive Cancer Center, Duarte, CA, USA; Department of Oncology, Providence St Jude Medical Center, Fullerton, CA, USA; Department of Medical Oncology, City of Hope Comprehensive Cancer Center, Duarte, CA, USA

**Keywords:** prostate cancer, relugolix, compliance, combination therapy, androgen deprivation therapy

## Abstract

**Background:**

Relugolix is the newest form of androgen deprivation therapy (ADT) approved for prostate cancer. However, as an oral drug, several real-world concerns exist, particularly medication compliance, safety with other androgen receptor-targeted agents, and financial burden to patients.

**Methods:**

A single institution retrospective chart review was conducted evaluating all patients who were prescribed relugolix for any prostate cancer indication from January 1, 2021 to January 31, 2022. Demographic data, cardiac risk factors, concomitant therapy usage, and PSA/testosterone levels, were abstracted from the chart review. Adverse effects were obtained by examining progress notes. Compliance was assessed by clinic notes as well as prescription fills by specialty pharmacy records. The reasons patients did not fill or discontinued the medication were noted.

**Results:**

Hundred and one patients were prescribed relugolix, and 91 patients consented to research. Seventy-one (78%) patients filled the prescription to relugolix, with a median follow-up of 5 months. Prescription fill data were available for 45 (63%) patients, with 94% of days covered. The most commonly reported reason not to fill was cost at 50%. Sixty-six (93%) patients reported never missing a dose. PSA levels were available in 71 (100%) patients with 69 (97%) showing stable or improved PSA. Testosterone levels were available in 61 (86%) of patients, which showed 61 (100%) stable or successful castration. Twenty-four (34%) patients used relugolix in combination. No new major safety signals were seen in combination therapy. Nineteen (27%) patients had switched to another form of ADT. Fifteen of these (79%) felt similar or better on relugolix therapy.

**Conclusions:**

Compliance with relugolix seemed acceptable. No major new safety signals were seen, even in combination. Among patients who switched therapy, most tolerated relugolix similarly or better than the previous form of ADT. The cost was a major reason for patients not initiating and for discontinuing therapy.

## Teaser

Relugolix combined with other standard prostate cancer therapies did not exhibit unexpected side effects. Compliance was good but some patients were unable to access this agent due to cost.

**Implications for Practice**

Relugolix is the newest form of androgen deprivation therapy approved for prostate cancer. From a high-volume center and early adopters of relugolix therapy, the authors share their experience with this novel drug, including some of the issues encountered with it. This retrospective study highlights practical and real-world issues encountered with relugolix. Concerns of compliance, data on combination therapy, and transitioning from either leuprolide or degarelix to relugolix are addressed.

## Introduction

The luteinizing hormone-releasing hormone (LHRH) analog leuprolide provides safe, reversible, and effective means for testosterone suppression in prostate cancer.^1^ By virtue of its mechanism of action, however, leuprolide initially raises testosterone levels before achieving castration, potentially leading to symptomatic flare in advanced disease.^2^ The LHRH antagonist degarelix was developed in part to avoid this, achieving faster, safer, and more consistent testosterone suppression.^3-8^

While both leuprolide and degarelix are generally well tolerated, their administration typically requires an injection in the physician’s office. Relugolix is an oral highly selective LHRH antagonist given daily that has been shown to achieve androgen deprivation in both phase I^9^ and phase II^10^ trials and was finally approved by the FDA in the phase III HERO trial.^2^ The HERO trial enrolled patients with castration-sensitive prostate cancer and compared their sustained castration rate at 48 weeks to leuprolide. While the safety profile of the 2 agents was similar, relugolix achieved castrate levels of testosterone faster and provided a higher overall rate of castration.^2^

Though the HERO trial revealed promising results for relugolix, it raised numerous practical questions, not least of which is the compliance concern. As an oral agent with rapid reversibility, it has the inherent risk of relying on patients to adhere to therapy, which maintains the suppression of testosterone. Compliance was facilitated during the trial with special pill bottles that issued audible reminders, which is not part of the commercial supply. While oral medications for prostate cancer are already being used, in our experience, there is variable adherence. Since missing doses of relugolix could result in testosterone breakthroughs, the real-world experience was needed to determine how well compliance and efficacy were maintained in a less-controlled setting. Moreover, the HERO trial did not address the experience of transitioning from injectable therapy to oral therapy, so evaluation of testosterone and PSA levels during this time will be informative to see if any rapid reversal or flare of testosterone is noted. In addition, while relugolix theoretically has similar efficacy to degarelix, adverse effects (AE) and financial costs need to be evaluated to better establish its place in the therapeutic armamentarium. Of note, the HERO trial used relugolix primarily as monotherapy without the additional androgen receptor (AR)-targeted therapy (ie, abiraterone and enzalutamide). While formal prospective combination trials are underway, we sought to detect any safety concerns from our early experience when using relugolix with concomitant oral (AR targeted) agents.

## Methods

An internal review board exemption was obtained for a single institution retrospective study. All patients who were prescribed relugolix from January 1, 2021 to January 31, 2022, and who had previously agreed to chart review, were identified using the electronic medical record system Epic via its SlicerDicer tool. Using Honest Broker, we screened out all patients who had refused participation in retrospective chart review research. Each patient was given a unique study identifier.

Demographic data included race/ethnicity, age at the time of prescription, and insurance provider. Insurance information was tabulated as Medicare, private insurance, Medi-Cal, and others.

Information about patients’ prostate cancers was examined; both indication of treatment and details on castration history were noted. Indication of treatment was defined as adjuvant setting, biochemical recurrence (BCR), metastatic disease-castration sensitive (mCSPC), and metastatic disease-castration resistant (mCRPC). Castration history was defined as castration naive, a previously castrated but new start of castration, switch from degarelix, and switch from leuprolide.

Concomitant therapy with other modalities was also tabulated. We recorded the use of any oral or infusional approved prostate cancer therapy that overlapped with the period during which relugolix was prescribed.

Cardiac risk factors were also tabulated and included coronary artery disease, diabetes, hypertension, hyperlipidemia, former or active smoking status, and body mass index (BMI) greater than 30 at treatment initiation.

Compliance was recorded based on documentation from a primary oncologist, and chart review. Using our institution’s specialty pharmacy dispensary records, we corroborated prescription fills where available.

Adverse events (AE) were collected primarily based on chart review. Objective information such as recorded weights and laboratory findings were reviewed and obtained where available. Chart text was searched using keywords related to the most frequent AE seen in the HERO trial including hot flashes, fatigue, diarrhea, constipation, pain, weight gain, urinary issues, behavioral changes, and cardiovascular events. Chart texts were also examined for any reports of unexpected or novel AE.

The rationale for either refusal to fill the prescription, or discontinuation, relugolix was obtained.

For patients who transitioned from either degarelix or leuprolide, the physician inquired whether relugolix was easier or more difficult to tolerate than prior injection therapy in terms of side effects. Outcomes of therapy continuation were also noted.

## Results

A total of 101 patients were identified who were prescribed relugolix in our institution from January 1, 2021 to January 31, 2022. Of those, 91 patients had agreed to retrospective research via Honest Broker.

Table 1 shows demographic data. Race and ethnicity showed 66 (72%) White, 6 (7%) Hispanic/Latino, 3 (3%) Black, 9 (10%) Asian, 1 (1%) Indian/Native, and 6 (7%) as not recorded. Age breakdown of 12 (23%) under the age of 65, 49 (54%) between the age of 65 and 74, and 21 (23%) over the age of 75. Regarding insurance, 59 (65%) had Medicare, 25 (27%) had private insurance, 5 (5%) had Medi-Cal, and 2 (2%) had other insurance. For comorbidities, 31 (34%) had a history of coronary disease, 16 (18%) had diabetes, 57 (63%) had hypertension, 46 (51%) had hyperlipidemia, 19 (21%) were former smokers, 2 (2%) were active smokers, and 25 (27%) had a BMI ≥30.

**Table 1.** Patient characteristics.

Characteristics	Total population, 91, *n* (%)	Filled relugolix, 71, *n* (%)
Race		
White-non-Hispanic	66 (73)	53 (75)
Hispanic/Latino	6 (7)	5 (7)
Black	3 (3)	3 (4)
Asian	9 (10)	5 (7)
American Indian/native American	1 (1)	1 (1)
Not reported	6 (7)	4 (6)
Insurance		
Medicare	59 (65)	43 (61)
Private	25 (27)	21 (30)
Medi-Cal	5 (5)	5 (7)
Other	2 (2)	2 (3)
Age		
<65	21 (23)	18 (25)
≥65, <75	49 (54)	39 (55)
≥75	21 (23)	14 (20)
Cardiac risk factors		
Coronary disease	31 (34)	23 (32)
Diabetes	16 (18)	14 (20)
Hypertension	57 (63)	41 (58)
Hyperlipidemia	46 (51)	33 (46)
Former smoker	19 (21)	16 (23)
Active smoker	2 (2)	2 (3)
BMI ≥30	25 (27)	21 (30)
Indication		
Adjuvant	22 (24)	18 (25)
BCR	37 (41)	27 (38)
mCSPC	24 (26)	19 (27)
Mcrpc	8 (9)	7 (10)
Castration history		
Castration naïve	36 (40)	31 (44)
Previously castrated	27 (30)	21 (30)
Switch degarelix	11 (12)	6 (8)
Switch leuprolide	17 (19)	13 (18)
Any switch	28 (31)	19 (27)
Follow-up	Median: 5 months	Range: 1-12 months

Abbreviations: BCR, biochemical recurrence; BMI, body mass index; mCSPC, metastatic disease-castration sensitive; mCRPC, metastatic disease-castration resistant.

All indications for androgen deprivation therapy (ADT) in prostate cancer were represented in the population. Twenty-two (24%) were prescribed relugolix as adjuvant therapy, 37 (40%) received relugolix therapy for BCR, 24 (26%) received relugolix therapy for mCSPC, and 8 (9%) received relugolix therapy for mCRPC. Thirty-six (40%) were newly castrated, 27 (30%) had a history of castration but were starting a new course of therapy, 11 (12%) were switched from active degarelix therapy, and 17 (19%) were switched from active leuprolide therapy. The median follow-up time was 5 months, with a range of 1-12 months.

Supplementary Table S1 shows available compliance data. 66 (93%) reported compliance in chart review and never missing a dose, while 5 (7%) did report missing at least one dose. This was corroborated by our institution’s specialty pharmacy fill records, which were available in 45 (63%) of our population. In these patients, the proportion of days covered by therapy was 94%. The range of prescription fills was 1-14, with a median of 4 fills. PSA levels were available in all 71 patients (100%), with 69 (97%) showing stable or decreased PSA values. The increase in PSA in the remaining 2 patients was attributed to progressive disease. Testosterone levels were available in 61 patients (86%), with all 61 (100%) showing stable or successful castration.

**Figure 1.**
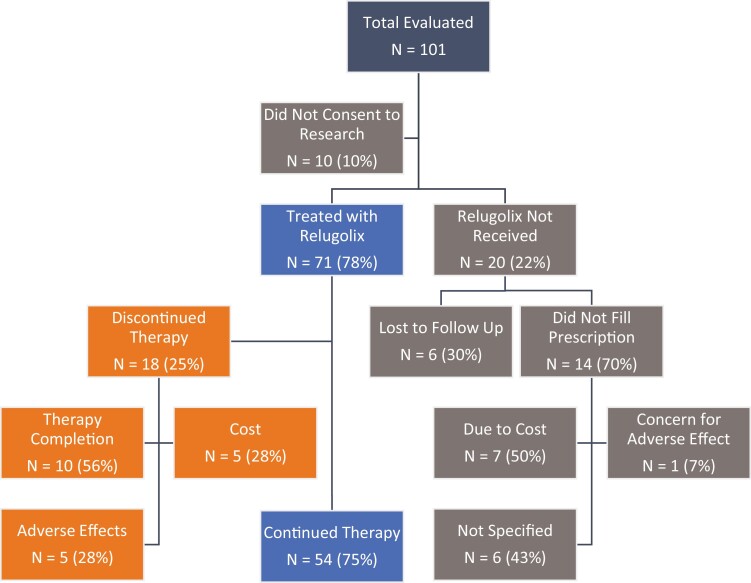
Consort diagram of patients evaluated from January 1, 2021 to January 31, 2022.

Outcomes, after prescription was issued for relugolix, are shown in Fig. 1. The cost was a major factor in failure to fill and discontinuation of relugolix. Fourteen patients (15%) never filled the prescription for relugolix, and 6 (7%) were lost to follow-up. Of the 14 who did not fill the prescription, 7 (50%) named cost as the primary reason, 1 (7%) named concern for AE, and 6 (43%) had other reasons. Subgroup analysis of the 14 patients who did not fill the prescription for relugolix is shown in Supplementary Table S2. Racial data demonstrates 9 (64%) White men, 4 (29%) Asian men, and 1 (7%) Hispanic men did not fill the prescription. Insurance subgroup analysis revealed that 11 (79%) of these men had Medicare, while 3 (21%) had private insurance.

Eighteen (25%) discontinued relugolix. The rationale behind discontinuation, with some overlapping reasons, was primarily found to be due to the completion of the prescribed therapy course (56%), while 5 patients (28%) discontinued due to cost, and 5 (28%) due to AE.

Table 2 shows that relugolix was used as monotherapy in 48 (68%) of patients. Of the patients who received combination 23 (32%), with some overlap enzalutamide was the most frequently co-prescribed agent with 10 patients (43%), abiraterone was the 2nd most common with 8 (35%), bicalutamide with 4 (17%), apalutamide with 3 (13%), and 1-2 patients each received relugolix with non-hormonal agents: docetaxel (4%), cabazitaxel (9%), and radium 223 (4%).

**Table 2.** Concomitant therapy.

Therapy	*n* (%)
Combination therapy	
Monotherapy	47 (66)
Combination	24 (34)
Therapeutic agent	
Bicalutamide	4 (17)
Abiraterone	8 (35)
Enzalutamide	10 (43)
Apalutamide	3 (13)
Docetaxel	1 (4)
Cabazitaxel	2 (9)
Radium 223	1 (4)

The AE profile is shown in Table 3. Commonly documented AE included hot flashes in 28 patients (39%), fatigue 18 (25%), urinary 13 (18%), behavioral 13 (18%), weight gain 11 (15%), pain 10 (14%), diarrhea 4 (6%), and constipation 0 (0%). and There were 5 (7%) major adverse cardiac events (MACE): myocardial infarction 1 (1%), cerebrovascular accident 2 (3%), revascularizations 2 (3%), and heart failure hospitalization 1 (1%). Patients who were on concomitant therapy reported an increased rate of fatigue 9 (39%), pain 7 (30%), weight gain 5 (22%), and decreased rate of behavioral issues 1 (4%) compared to monotherapy: 9 (19%), 3 (6%), 6 (13%), and 11 (23%), respectively. No novel or unexpected AE was documented in patients receiving combination therapy.

**Table 3.** Adverse effects.

**Total (*N*** **=** **71)**		**%**	**Mono** **(*N*** **=** **47)**	**%**	**Combo** **(*N*** **=** **24)**	**%**	**Bica** **(*N*** **=** **4)**	**%**	**Abi** **(*N*** **=** **8)**	**%**	**Enza** **(*N*** **=** **10)**	**%**	**Apa** **(*N*** **=** **3)**	**%**	**Doce** **(*N*** **=** **1)**	**%**	**Cab** **(*N*** **=** **2)**	**%**	**Radium** **(*N*** **=** **1)**	**%**
** **Hot flash	28	39	19	40	9	38	1	25	3	38	4	40	1	33	0	0	0	0	1	100
** **Fatigue	18	25	9	19	9	38	1	25	4	50	3	30	1	33	1	100	1	50	1	100
** **Diarrhea	4	6	3	6	1	4	0	0	0	0	1	10	0	0	0	0	0	0	0	0
** **Const	0	0	0	0	0	0	0	0	0	0	0	0	0	0	0	0	0	0	0	0
** **Pain	10	14	3	6	**7**	29	1	25	**3**	38	3	30	1	33	1	100	1	50	**0**	0
** **Weight gain	11	15	6	13	5	21	1	25	3	38	1	10	0	0	0	0	0	0	1	100
** **Urinary	13	18	12	26	1	4	0	0	0	0	1	10	0	0	0	0	0	0	0	0
Adverse effects MACE																				
Total (*N* = 71)		%	Mono (*N* = 47)	%	Combo (*N* = 24)	%	Bica (*N* = 4)	%	Abi (*N* = 8)	%	Enza (*N* = 10)	%	Apa (*N* = 3)	%	Doce (*N* = 1)	%	Cab (*N* = 2)	%	Radium (*N* = 1)	%
Total w/MACE	5	7	4	9	1	4	0	0	0	0	1	10	0	0	0	0	0	0	0	0
MI	1	1	1	2	0	0	0	0	0	0	0	0	0	0	0	0	0	0	0	0
CVA/TIA	2	3	1	2	1	4	0	0	0	0	1	10	0	0	0	0	0	0	0	0
Revascularization	2	3	2	4	0	0	0	0	0	0	0	0	0	0	0	0	0	0	0	0
HF event	1	1	1	2	0	0	0	0	0	0	0	0	0	0	0	0	0	0	0	0

Abbreviations: Abi, abiraterone; Apa, apalutamide; Bica, bicalutamide; Cab, cabazitaxel; Combo, combination therapy; CVA/TIA, cerebral vascular accident/transient ischemic attack; Doce, docetaxel; HF event, heart failure hospitalization; MACE, major adverse cardiac events; MI, myocardial infarction; Mono, monotherapy; Radium, Radium 223.

**Figure 2.**
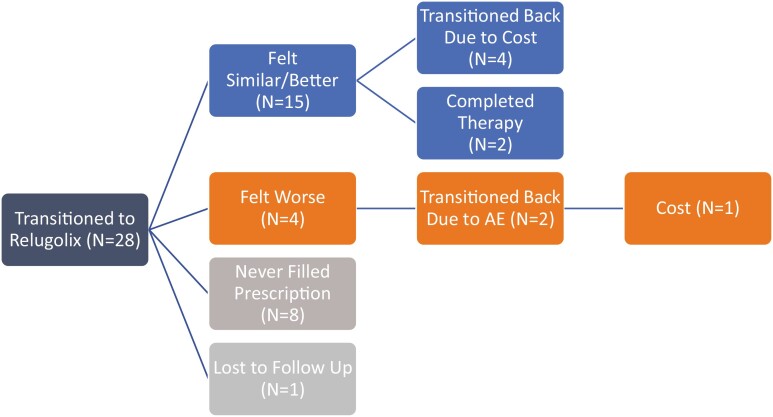
Patients who switched from either leuprolide or degarelix to relugolix and their therapy outcomes. One patient switched back to previous therapy due to cost and adverse effects. Abbreviation: AE, adverse effects.

Fig. 2 shows the outcomes of the 28 patients (31%) who switched from another form of ADT. Of these 8 (29%) never filled the prescription. Of the remaining 19 patients, 15 (79%) felt similar or better than their previous form of ADT while 4 (21%) felt worse on relugolix. Of the patients who felt similar, 6 discontinued therapies as 4 (66%) switched back due to cost and 2 (33%) due to therapy completion. Of those who felt worse, 2 (50%) discontinued therapy, both due to AE, while 1 also cited cost. In 8 patients with available testosterone levels before and after the switch, there was no change observed in all 8 patients (100%). In 15 patients with PSA levels available from before and after the switch, all 15 had stable levels or exhibited continued decline (Supplementary Table S3).

## Discussion

The HERO trial led to the approval of relugolix, the first orally available form of LHRH manipulation for the treatment of prostate cancer.^2^ While the trial did show that relugolix was safe and highly effective in treating prostate cancer, some real-world and practical concerns were raised, particularly around compliance and financial implications, since prescription drug coverage can be less robust than primary insurance portions covering clinic visits and injection medications. Our retrospective study evaluating our institution’s first 91 patients who filled the prescribed relugolix shows that cost significantly impacted access to this medication. Borrelli et al. estimate the yearly average out-of-pocket cost for Medicare patients of relugolix to be $3731, compared to $2912 for leuprolide.^11^ Financial toxicity is becoming a more widely discussed topic in modern American medicine but remains incompletely understood.^12^ Carrera et al. explains that the advent of new technology and medications can come at a variable financial burden, particularly in the USA where we have access to more antineoplastic therapeutics and a wide range of insurance plans. Higher out-of-pocket costs can lead not only to more psychosocial stress but can even result in drug abandonment. However, oral therapy could alleviate other financial constraints such as time off work to travel to the clinic for injections. Interestingly, while the power was low to draw definitive conclusions, those with Medicare insurance represented the largest proportion of patients who refused the fill the prescription of relugolix. Medicare patients often encounter significant financial burdens, especially when dealing with oral antineoplastic therapeutics. Regarding private insurance, pharmaceutical companies can offer direct-to-patient assistance, resulting in reduced copays, and easier access to relugolix. Those with Medi-Cal insurance did not have any refusal to fill suggesting that this population has good access to relugolix already, likely in part as they do not have to deal with coverage gaps in Medicare part D. Further subgroup analysis of race showed a larger proportion of White and Asian men who refused to fill relugolix, compared to Hispanic, Black, and Native Americans. Our analysis was not able to compare individual out-of-pocket costs with income, so whether there is a financial burden differential versus a potential cultural preference for injection therapy may need to be explored. Culturally tailored educational materials about the medication and accessing copay relief may need to be created to support patients in their decision-making.

In those who were able to obtain relugolix, compliance appears to be acceptable, despite the lack of specialized pill bottles which were used in the HERO study. Only a few patients reported missing doses, and the available specialty pharmacy data indicated that there were no issues with prescription fills. Furthermore, both PSA and testosterone levels indicated stable or improved disease states, supporting stable castration. The high rate of compliance is not completely unexpected, and akin to other oral neoplastic therapy available in oncology,^13^ however, this is with the caveat that our study involved patients motivated enough to receive care at a comprehensive cancer center and may not reflect the compliance of a broader community oncology patient population. Regardless, it is reassuring to see that no significant compliance challenges were reported in this population during relugolix therapy. Even among those who missed doses, including those who missed regularly, available PSA and testosterone levels remained low. This is of particular interest given relugolix’s rapid reversal of castration, which raised the concern that the short half-life of relugolix might lead to testosterone breakthrough in patients who missed doses. While patients should be encouraged to take doses as close as possible to the same time each day without missing doses, and all best practices to support oral therapy compliance should be brought to bear, providers can reassure patients upset about having missed doses that it is unlikely to result in testosterone breakthrough based on our early experience.

A similar toxicity profile was seen in our patient population compared to the HERO trial, with no additional novel or unexpected AE. However, our patients tended to complain less of hot flashes, and gastrointestinal issues suggesting that these AE while present may be manageable. Though no major safety signals excluding MACE were seen, patients on relugolix often complained about urinary issues, weight gain, and behavioral changes. These differences may be due to the retrospective nature of our data collection relying on what was both reported and documented in the clinical record, or due to the inclusion of patients with castration-resistant disease who may have had longer overall exposure to castration and additional systemic therapies. Relatively more cardiovascular events were seen in patients during our study period compared to historical controls though this likely is attributable to the relatively small sample size. The PRONOUNCE trial compared cardiovascular risk in patients treated with degarelix and leuprolide and found that in a study of 545 patients, 4.1%-5.5% of patients experienced cardiovascular events,^14^ similar to what was observed in our study as well.

Our findings support the safety data reported in HERO, and further expand on the safety of relugolix, including in patients with castration-resistant disease, patients switching from an injection LHRH therapy, and patients taking relugolix in combination with other approved prostate cancer therapies. Though the safety data with relugolix combination is reassuring, prospective studies are needed to provide more definitive evidence of toxicity profiles with various AR-targeted agents. Combination use should still be taken with great caution outside of a clinical trial.

Subgroup analysis of patients who switched from another form of ADT showed that they tended to tolerate relugolix as well as, or even better than their previous ADT agent. Among those who felt worse, AE was not profound enough to warrant switching back indicating that the convenience of oral therapy contributed to the overall improved quality of life patients’ experiences. The benefit was seen more clearly in patients switching from GnRH agonism which likely is attributable to the mechanistic differences to GnRH antagonism. Regardless of which ADT injection patients are on, a trial of relugolix could be considered in those who experience unacceptable AE. All patients with available PSA and testosterone showed that they had the stable or improved disease during the transition to relugolix therapy alleviating the fear of disease flare during the transition.

While our data has the advantage of highlighting real-world practice, there are several limitations to consider. As a retrospective study, rigorous collection of compliance and AE data was not possible. This information is often collected passively, and even if discussed, the documentation does not always fully reflect what is discussed in a patient encounter. Furthermore, this study represents a single institution experience in an academic medical oncology setting and may not reflect all populations of prostate cancer patients. Finally, granular data about cost and decision-making could not be obtained, and would be important to shed light on how impactful the financial aspects of the prescription were compared to other concerns.

## Conclusion

Relugolix is the first oral LHRH analogue, approved due to the safety and efficacy demonstrated in the HERO trial. Our early commercial experience shows that while medication compliance does not appear to be a major issue, the cost did impact how many patients filled their prescriptions or discontinued relugolix. No new safety signals were observed in patients taking relugolix in combination with AR-targeted agents or switching from injection therapy.

## Supplementary Material

oyad036_suppl_Supplementary_Table_S1

oyad036_suppl_Supplementary_Table_S2

oyad036_suppl_Supplementary_Table_S3

## Data Availability

The data underlying this article will be shared on reasonable request to the corresponding author.
