# Supplementary material for: A Practical Guide to Relugolix: Early Experience With Oral Androgen Deprivation Therapy
Source: Oncologist. 2023 Mar 8;28(8):699–705. doi: 10.1093/oncolo/oyad036 (PMC10400130; doi:10.1093/oncolo/oyad036)
Supplement: oyad036_suppl_Supplementary_Table_S1 [file oyad036_suppl_supplementary_table_s1.pdf]

Compliance Data		
Reporting Missing Dose	N	%
Yes	66	93%
No	5	7%
PSA	N	%
Available	71	100%
Stable/Decreased	69	97%
Increase	2	3%
Testosterone	N	%
Available	61	86%
Stable/Improved Castration	61	100%
Loss of Castration	0	0%
Pharmacy Data		
Available	N = 45	63%
Proportion of Days Covered	94%	
Number of Prescription Fills	Median: 4	Range: 1-14
