# Supplementary material for: A Practical Guide to Relugolix: Early Experience With Oral Androgen Deprivation Therapy
Source: Oncologist. 2023 Mar 8;28(8):699–705. doi: 10.1093/oncolo/oyad036 (PMC10400130; doi:10.1093/oncolo/oyad036)
Supplement: oyad036_suppl_Supplementary_Table_S2 [file oyad036_suppl_supplementary_table_s2.pdf]

Patients Who Did Not Fill Relugolix Prescription		
Race	**N**	**%**
White – Non-Hispanic	9	64%
Hispanic/Latino	1	7%
Black	0	0%
Asian	4	29%
American Indian/Native	0	0%
Not Reported	0	0%
Insurance	N	%
Medicare	11	79%
Private	3	21%
Medi-Cal	0	0%
Other	0	0%
