# Supplementary material for: A Practical Guide to Relugolix: Early Experience With Oral Androgen Deprivation Therapy
Source: Oncologist. 2023 Mar 8;28(8):699–705. doi: 10.1093/oncolo/oyad036 (PMC10400130; doi:10.1093/oncolo/oyad036)
Supplement: oyad036_suppl_Supplementary_Table_S3 [file oyad036_suppl_supplementary_table_s3.pdf]

Available PSA and Testosterone Levels by 3 Months in Patients Who Switched to Relugolix		
PSA	**N**	**%**
Available	15	79%
Stable/Decreased	15	100%
Increased	0	0%
Testosterone	**N**	**%**
Available	9	47%
Castration Stable/Achieved	100	100%
Loss of Castration	0	0%
